# LncRNA PRADX-mediated recruitment of PRC2/DDX5 complex suppresses UBXN1 expression and activates NF-κB activity, promoting tumorigenesis

**DOI:** 10.7150/thno.54549

**Published:** 2021-03-04

**Authors:** Yansheng Li, Xing Liu, Xiaoteng Cui, Yanli Tan, Qixue Wang, Yunfei Wang, Can Xu, Chuan Fang, Chunsheng Kang

**Affiliations:** 1 Department of Neurosurgery, Tianjin Medical University General Hospital, Laboratory of Neuro-oncology, Tianjin Neurological Institute, Key Laboratory of Post-Neurotrauma Neuro-Repair and Regeneration in Central Nervous System, Ministry of Education and Tianjin City, Tianjin 300052, China.; 2 Department of Breast Surgery, The Affiliated Yantai Yuhuangding Hospital of Qingdao University, 20 Yudong Road, Yantai, Shandong 264001, P.R. China.; 3 Beijing Neurosurgical Institute, Department of Neurosurgery, Beijing Tiantan Hospital, Capital Medical University, Beijing 100050, China.; 4 Department of Pathology, Affiliated Hospital of Hebei University, Baoding 071000, China.; 5 Department of Neurosurgery, Hebei University affiliated Hospital, Baoding 071000, China.

**Keywords:** PRADX, long noncoding RNA, glioblastoma, colon adenocarcinoma, NF-κB, TCGA, Polycomb repressive complex 2, DEAD box protein 5.

## Abstract

**Rationale:** Accumulating evidence indicates that long noncoding RNAs (lncRNAs) play crucial roles in cancer progression; however, only few have been characterized in detail. The current study aimed to identify a novel cancer driver lncRNA in glioblastoma and colon adenocarcinoma.

**Methods:** We performed whole transcriptome analysis of TCGA pan-cancer datasets to compare the lncRNA expression profiles of tumor and paired normal tissues. *In situ* hybridization of tissue sections was performed to validate the expression data and determine the localization of lncRNAs that may be linked to glioblastoma and colon adenocarcinoma. Chromatin isolation by RNA purification (ChIRP), chromatin immunoprecipitation (ChIP), and Co-immunoprecipitation (Co-IP) assays were performed to assess the interaction between lncRNA, proteins, and chromatin. The functional significance of the identified lncRNAs was verified *in vitro* and *in vivo* by knockdown or exogenous expression experiments.

**Results:** We found a lncRNA ENST00000449248.1 termed PRC2 and DDX5 associated lncRNA (PRADX) that is highly expressed in glioblastoma and colon adenocarcinoma cells and tissues. PRADX, mainly located in the nucleus of tumor cells, could bind to EZH2 protein via the 5' terminal sequence. Moreover, PRADX increased the trimethylation of H3K27 in the UBXN1 gene promoter via PRC2/DDX5 complex recruitment and promoted NF-κB activity through UBXN1 suppression. Knockdown of PRADX significantly inhibited tumor cell viability and clonogenic growth *in vitro*. In xenograft models, PRADX knockdown suppressed tumor growth and tumorigenesis and prolonged the survival of tumor-bearing mice.

**Conclusions:** PRADX acts as a cancer driver and may serve as a potential therapeutic target for glioblastoma and colon adenocarcinoma.

## Introduction

Multi-step changes in the genome promote cancer progression. Around 70% of the human genome is transcribed into RNA, whereas less than 2% is protein coding 1-3. Thus, there are large numbers of noncoding RNAs that are encoded by human genome. Accumulating evidences indicate that these noncoding RNAs are strongly associated with multiple cellular processes ranging from development to diseases 4-7. Long noncoding RNAs (lncRNAs) are defined as non-protein coding transcripts that are longer than 200 nucleotides 1, 3. The functional role of lncRNAs involves diverse cellular and molecular mechanisms. For instance, lncRNAs serve as scaffolds or guides for regulating protein-protein or protein-DNA interaction, as decoys for binding to protein or microRNAs, and as enhancers to promote gene transcription 8-11. Moreover, multiple studies have shown that lncRNAs are abnormally expressed in a variety of tumors and act as either tumor suppressor or oncogene 12-14. Notably, lncRNAs have been shown to interact with chromatin-modifying complexes, such as Polycomb repressive complex 2 (PRC2), and guide epigenetic regulations in various types of cancers 15, 16.

Polycomb repressive complex 2 is a multi-protein chromatin modifying complex and comprises of the following core catalytic components: suppressor of zeste 12 homolog (SUZ12), enhancer of zeste homologue 2 (EZH2) and embryonic ectoderm development protein (EED) 17, 18. The complex catalyzes the addition of up to three methyl groups on histone H3 at lysine 27 (H3K27me3); thus, it promotes tumorigenesis by suppressing tumor suppressors 19. A recent study suggested that DEAD box protein 5 (DDX5) plays a significant role in regulating the stability and function of PRC2 20. Interestingly, knockdown of DDX5 causes reduced occupancy of SUZ12 and EZH2, and with reduced H3K27me3 occupancy in hepatocellular carcinoma cells 20. However, the role of DDX5 in lncRNA-mediated chromatin modification is poorly understood.

The large-scale whole-genome technologies accelerate our understanding of human cancer and the development of molecular targeted therapies. For a decade, The Cancer Genome Atlas (TCGA) project has collected clinicopathological data along with multi-platform molecular profiles of more than 11,000 human samples across 33 different types of cancer 21.

Here, we performed whole transcriptome analysis of TCGA pan-cancer datasets to discover the distinct lncRNA profiling between tumors and paired normal tissues. Further, we identified and characterized a novel lncRNA, ENST00000449248.1 (PRADX) that was found to be upregulated in glioblastoma (GBM) and colon adenocarcinoma (COAD), and predominantly localized in the nucleus of tumor cells. PRADX could bind to EZH2 protein via 1-500 bp 5' terminal sequence and increased H3K27me3 level on the UBXN1 gene promoter by recruiting the PRC2/DDX5 complex, resulting in UBXN1 suppression, which in turn promoted NF-κB activity. Inhibition of PRADX could suppress tumor growth and tumorigenesis, and prolong the survival of tumor bearing mice, indicating that PRADX acts as a cancer driver, and may serve as a potential therapeutic target.

## Methods and Methods

### Pan-cancer profiles and lncRNA screening

The pan-cancer transcriptomic (polyA+ IlluminaHiSeq) and clinical data corresponding to 33 tumor types were downloaded from TCGA data portal (https://portal.gdc.cancer.gov/). The lncRNAs were annotated using the human genome (GRCh38) GENCODE version 33 and Ensembl 99 (http://www.gencodegenes.org). Tumor types with at least 20 adjacent normal samples were used for differential screening of the lncRNAs. The lncRNAs differentially expressed with an adjusted *P* value of < 0.05 were considered statistically significant and were used for subsequent analysis. Basic details of lncRNAs, their possible relationship with types of cancers and the co-expression networks were obtained from the Lnc2Catlas database (https://lnc2catlas.bioinfotech.org) 22.

### Cell lines and cell culture

Patient-derived primary glioblastoma N33 cell line was provided by Professor Fan (Beijing Key Laboratory of Gene Resource and Molecular Development, Laboratory of Neuroscience and Brain Development, Beijing Normal University) and has been reported in our previous study 23. N33 cells were cultured in Dulbecco's Modified Eagle's Medium (DMEM/F12, 1:1; Gibco) containing 10% fetal bovine serum (FBS). Human glioblastoma cell line, U87-MG, colon adenocarcinoma cell lines, SW480 and HT29 were purchased from American Type Culture Collection (ATCC). U87-MG and SW480 cells were cultured in DMEM with 10% FBS (Corning), whereas, HT29 cells were cultured in McCoy's 5A modified medium (Gibco) supplemented with 10% FBS. All the cells were incubated at 37 °C in 5% CO_2_.

### 5′ and 3′ Rapid Amplification of cDNA Ends (RACE)

1 μg of total RNA was used for 5′race and 3′race analysis. The SMARTer RACE cDNA Amplification Kit (Clontech, CA) was used according to the manufacturer's instructions. The gene-specific primers used for the PCR of the RACE analysis were 5′-ACGGTGGCCCAGGCTCCAAT-3′; 5′-GAAGCCAGGCGGTTGGAGAAACATT-3′ (5′RACE) and 5′-ACATCTGTTTCTGGGCATCCGCAC-3′; 5′-ACTCCATGCGGCTCCACGAGGT-3′ (3′RACE). RACE PCR products were separated on a 1% agarose gel. The gel extraction product was cloned into the pGH-T vector, and the indicator primers were used for bi-directional sequencing (Sagene, China).

### Cell transfection

Cells were seeded in 6-well plates at 70-80% confluence. To overexpress PRADX, the cells were transfected with plasmid containing PRADX sequence (Ibsbio, Shanghai, China) using Lipofectamine 3000 (Invitrogen, Carlsbad, CA, USA) according to the manufacturer's instructions. To knockdown the expression of PRADX, SUZ12, EZH2 or DDX5 genes, the cells were transfected with respective siRNAs (Ibsbio, Shanghai, China) using Lipofectamine 3000, as previously described 24. The siRNA sequences targeting each gene are shown in Table S1. Lentiviruses containing si-PRADX-1 sequence and negative control sequence were obtained from Ibsbio (Shanghai, China).

### Cell Counting Kit 8 (CCK-8) and clonogenic assays

Cell viability was evaluated using CCK-8 assay (Dojindo, Japan). A total of 3×10^3^ cells per well were seeded in 96-well plates and incubated for 24 h before the transfection. After 24, 48, 72, or 96 h of transfection, CCK8 was added to the culture, followed by 1 h incubation. The absorbance at 450 nm (OD450) was measured using the BioTek Gen5 Microplate Reader (BioTek Instruments, USA).

For the clonogenic assay, 6-well plates were seeded with 300 cells per well and incubated for two weeks till the colonies could be observed by naked eye. The cells were then fixed with 4% methanol and stained with 0.1% crystal violet. The number of colonies was captured by an Olympus camera (Tokyo, Japan) and counted by ImageJ.

### Co-Immunoprecipitation (Co-IP) and mass spectrometry (MS)

The cells were lysed using Western and IP lysis buffer (Beyotime Biotechnology) and the cell lysate was incubated with 40 μL of protein-A/G agarose beads (Millipore), 5 μg anti-EZH2, anti-SUZ12 (Cell Signaling Technology, CST) or anti-DDX5 (proteintech#67025-1-Ig) antibodies at 4 °C overnight. After washing five times with phosphate buffered saline (PBS) buffer, the samples were analyzed by Western blotting or MS.

### Chromatin immunoprecipitation (ChIP) and ChIP-reChIP

ChIP experiments were performed using the Millipore Magna ChIP^TM^ A/G kit (catalog # 17-10085). Briefly, 6×10^6^ cells were cross-linked with 1% formaldehyde for 10 min and neutralized with 10× glycine for 5 min at room temperature. Next, the cell lysate was sonicated still the length of DNA was 200-1000 bp. An equal amount of chromatin was immunoprecipitated at 4°C overnight with 5 μg of the following antibodies: H3K27me3, EZH2, histone H3, DDX5, SUZ12 and RNA Pol II phospho-Ser2 (CST). Immunoprecipitated products were collected after incubation with Magnetic Beads Protein A/G. The primers used for UBXN1 have been described elsewhere 25.

ChIP-reChIP assay was performed following the previously described protocol 26. Sonicated chromatin was first immunoprecipitated with 1^st^ antibodies overnight and eluted by incubation at Re-ChIP elution buffer. Then, 2^nd^ antibodies were utilized to re-immunoprecipitate the correlated chromatin. The immunoprecipitated DNA was isolated and examined by PCR.

### Protein preparation and Western blotting

Total proteins were prepared from cells using pre-chilled RIPA buffer with proteinase and phosphatase inhibitor cocktails (Selleck.cn, Shanghai, China). The polyvinylidene fluoride (PVDF) membranes were incubated overnight at 4 °C with primary antibodies followed by incubation with horseradish peroxidase-labeled secondary antibody (Zsbio Store-bio, Beijing, China) at room temperature for 1 h. The protein bands were visualized using a chemiluminescence reagent (ECL) kit (Boster, Wuhan, China). The antibodies used are shown in Table S1.

### RNA isolation and real-time quantitative reverse-transcription PCR (RT-qPCR)

Total RNA from the cultured cells was extracted using TRIzol reagent (Invitrogen, USA). The nuclear and cytoplasmic fractions were separated using 0.5% NP-40 (Solarbio, Beijing, China) with an RNAase inhibitor (Promega, USA), followed by RNA extraction using TRIzol reagent (Sigma, USA). One microgram of the total RNA was used as template for cDNA synthesis using a PrimeScript RT Reagent Kit (Takara, Japan). Real-time quantitative PCR was performed in triplicate using the SYBR Green reaction mix (Takara, Japan) on a CFX96 Touch Real-Time PCR Detection System (Bio-Rad, USA). The primer sequences used for RT-qPCR are listed in Table S1.

### RNA immunoprecipitation (RIP)

RIP was performed using Magna RIP RNA-Binding Protein Immunoprecipitation Kit (Cat. # 17-701, Millipore, USA) according to the manufacturer's instructions. The EZH2 antibody used for RIP was purchased from CST. Further, the RNA fraction precipitated by RIP was analyzed using RT-qPCR.

### Confocal immunofluorescence microscopy

Cells were grown overnight on coverslips and then covered to a depth of 2-3 mm with 4% formaldehyde diluted in 1 × PBS for 15 min in room temperature. Then the fixative was aspirated, followed by rinsing thrice, each for 5 min, in 1 × PBS. After treating with 0.5% Triton-X100 (ThermoFisher, USA) diluted in warm PBS and blocking agent in blocking buffer (5% bovine serum albumin diluted in warm PBS, BioFroxx, Guangzhou, China) for 1 h at room temperature, the cells were incubated with primary antibodies overnight at 4 °C followed by secondary antibodies for 1 h at room temperature. The primary and secondary antibodies used are shown in Table S1. Nuclear staining was performed using 1 μg/mL of 4'-6-diamidino-2-phenylindole (DAPI, Molecular Probes, D1306). Protein subcellular localization was observed under a Zeiss 510 META or Leica TCS-SP2 confocal laser scanning microscope.

### RNA *in situ* hybridization histochemistry (ISH)

RNA ISH experiments were performed using the RNA *in situ* hybridization kit (Boster, China) according to the manufacturer's instructions as previously described 23. The probes (Table S1) were designed and synthesized by Boster (Wuhan, China). The cancer and adjacent normal tissues were surgically resected from patients at the Affiliated Hospital of Hebei University. The patients were diagnosed according to the histopathological evaluations, and no pre-operative treatment was conducted. Written informed consent was obtained from all the patients in accordance with the Declaration of Helsinki and the study was approved by the Clinical Research Ethics Committee of Hebei University. The quantification of PRADX in tissues was measured by ImageJ software.

### Chromatin isolation by RNA purification (ChIRP)

ChIRP was performed following the previously described protocol by Chu and Chang 27. The DNA probes designed against the PRADX full-length sequence (designed by Ibsbio, Shanghai, China) were biotinylated at the 3' end. The mock control used in the current experiment has been described in our previous study 24. The biotin probe sequences are listed in Table S1. RNA extraction was performed using TRIzol reagent to confirm the RNA enrichment. DNA samples were amplified by qPCR. Protein elution was performed by resuspending the beads in DNase buffer with a cocktail of 100 µg/mL RNase A, 0.1 U/µL RNase H and 100 U/mL DNase I at 37 °C for 30 min, followed by detection using Western blotting.

### Hematoxylin and eosin (H&E) staining and Immunohistochemistry (IHC)

Hematoxylin and eosin (H&E) staining, and IHC assays were performed as per the methods described in our previous study 23. The primary antibodies used in IHC are listed in Table S1. The quantification of UBXN1 in tissues was measured by ImageJ software.

### *In vivo* xenograft mouse models

BALB/c nude mice aged 4 weeks were purchased from Beijing Vital River Laboratory Animal Technology. To establish the GBM models, PRADX knockdown or control U87-MG cells (3 × 10^5^ cells per mouse in 3 μL PBS) transfected with luciferase lentivirus were injected into the intracranial. A parietal bioluminescence imaging using the IVIS Lumina Imaging System (Xenogen) was used to detect the tumor growth on day 14. In addition, survival of mice was monitored during the tumor progression and overall survival curves were generated using the Kaplan-Meier method. PRADX knockdown or control HT29 cells (3 × 10^6^ cells per mouse in 10 μL PBS) were injected into the subcutaneous space to establish the COAD models. Each experimental group comprised of 6 mice. The tumor volume was calculated using a caliper by the following formula: volume=length*width2. After death or euthanasia, the brains or subcutaneous tumor tissues were carefully extracted, formalin fixed, paraffin embedded and used for IHC analysis.

### Statistical analysis

Student's t-test was used for comparing the variables between two groups, whereas, one-way analysis of variance (one-way ANOVA) was used for the comparison of at least three groups. GraphPad Prism 8 was used for doing the statistical analysis. Comparisons of binary and categorical patient characteristics between the subgroups were performed using Chi-squared test. Cytoscape and Revigo 28 were used to visualize the significantly enriched Gene Ontology (GO) terms. Gene set enrichment analysis (GSEA) was performed for exploring the functional importance of PRADX. For prognostic analysis, the patients with an overall survival time of < 30 days were excluded to avoid the influence of perioperative death. Cox regression analysis was performed using R 3.6.0. The error bars in the figures represent mean ± SD from at least three independent experiments. A *P* value of < 0.05 was considered statistically significant.

## Results

### Whole transcriptome analysis revealed a distinct lncRNA profile between the tumor and matched normal tissues

**Figure 1:**
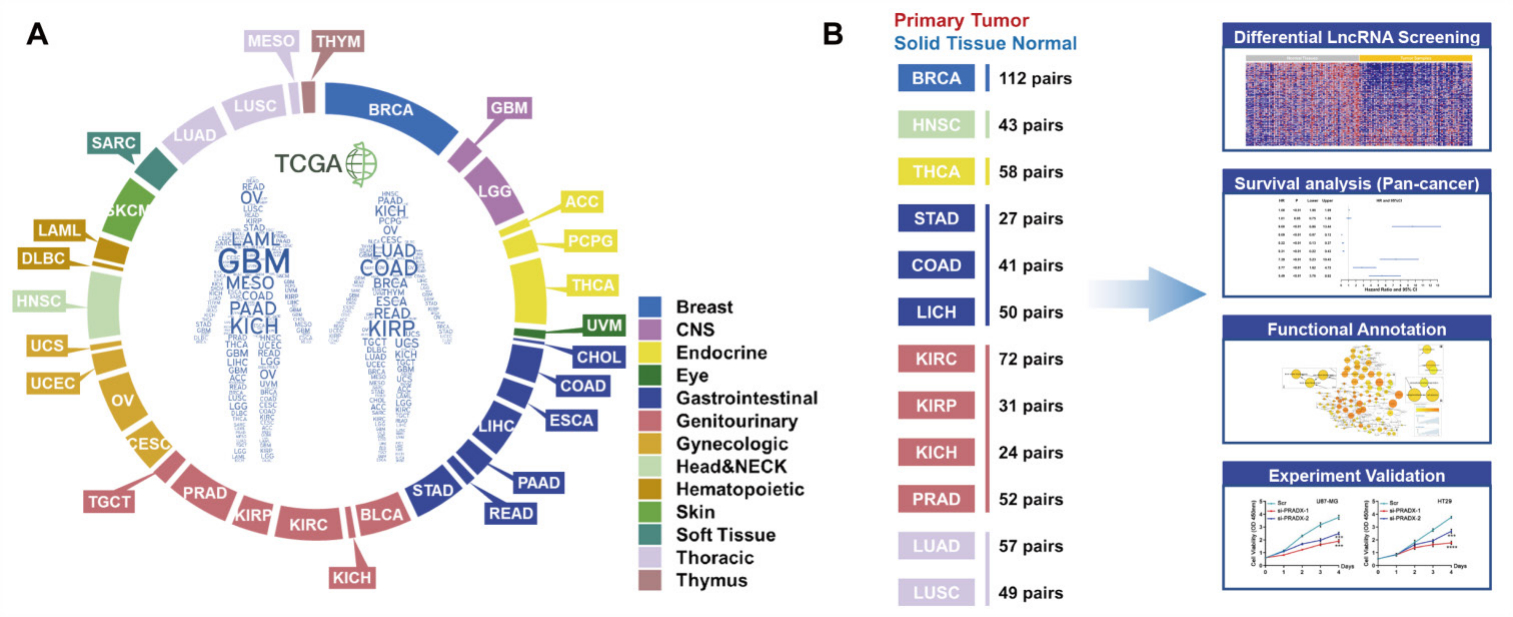
TCGA pan-cancer dataset and workflow. **A** Distribution of 33 cancer types across different tissue and organ systems. **B** Workflow describing the screening and functional exploration of the lncRNAs.

**Figure 2:**
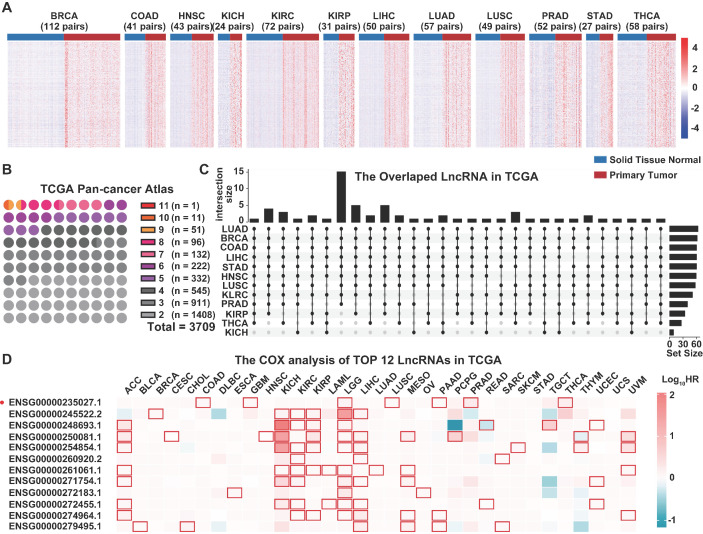
Screening of the dysregulated lncRNAs in TCGA pan-cancer atlas transcriptome data. **A** Differentially expressed lncRNAs between primary tumors and matched normal tissues. **B** Number of upregulated lncRNAs across multiple cancer types. **C** Upset diagram showing the number of shared lncRNAs upregulated in at least 10 cancer types. **D** Univariate COX analysis of the 12 shared lncRNAs in TCGA pan-cancer atlas.

The TCGA pan-cancer atlas contained a set of 9,560 samples from 9 body systems and 33 different cancer type (Figure 1A). The transcriptome data was downloaded from the TCGA pan-cancer atlas and annotated using the GENCODE version 33 to explore the role of lncRNAs in tumorigenesis. A total of 10,656 lncRNAs were identified across all the cancer types. In the pan-cancer dataset, 12 of the tumor types (BRCA, HNSC, THCA, STAD, COAD, LIHC, KIRC, KIRP, KICH, PRAD, LUAD and LUSC) had at least 20 tumor-adjacent normal samples (Figure 1B), and were used for the screening of differentially expressed lncRNAs between the cancer and normal tissues (Figure 2A, Table S2). We mainly focused on the upregulated lncRNAs that might be implicated in tumorigenesis. Furthermore, we identified the shared and exclusively upregulated lncRNAs across the 12 cancer types (Figure 2B-C, Table S3). Notably, no lncRNA was upregulated in all the 12 tumor types, whereas, 12 lncRNAs were upregulated in at least 10 cancer types. To investigate the prognostic implications of these lncRNAs, we correlated their expression with overall survival in 33 cancer types (Figure 2D, Table S4). Every selected lncRNA is statistically associated with overall survival of distinct tumor types. Among the 12 shared lncRNAs, ENST00000449248.1 (encoded by the gene ENSG00000235027.1), also termed as PRC2 and DDX5 associated lncRNA (PRADX), may serve as a prognostic indicator in GBM (HR=1.165, 95% CI=1.008-1.346, *P* < 0.05) and COAD (HR=1.1830, 95% CI=1.079-1.297, *P* < 0.001).

### PRADX, a novel lncRNA, is predominantly distributed in the nucleus of tumor cells and highly expressed in glioblastoma and colon adenocarcinoma

**Figure 3:**
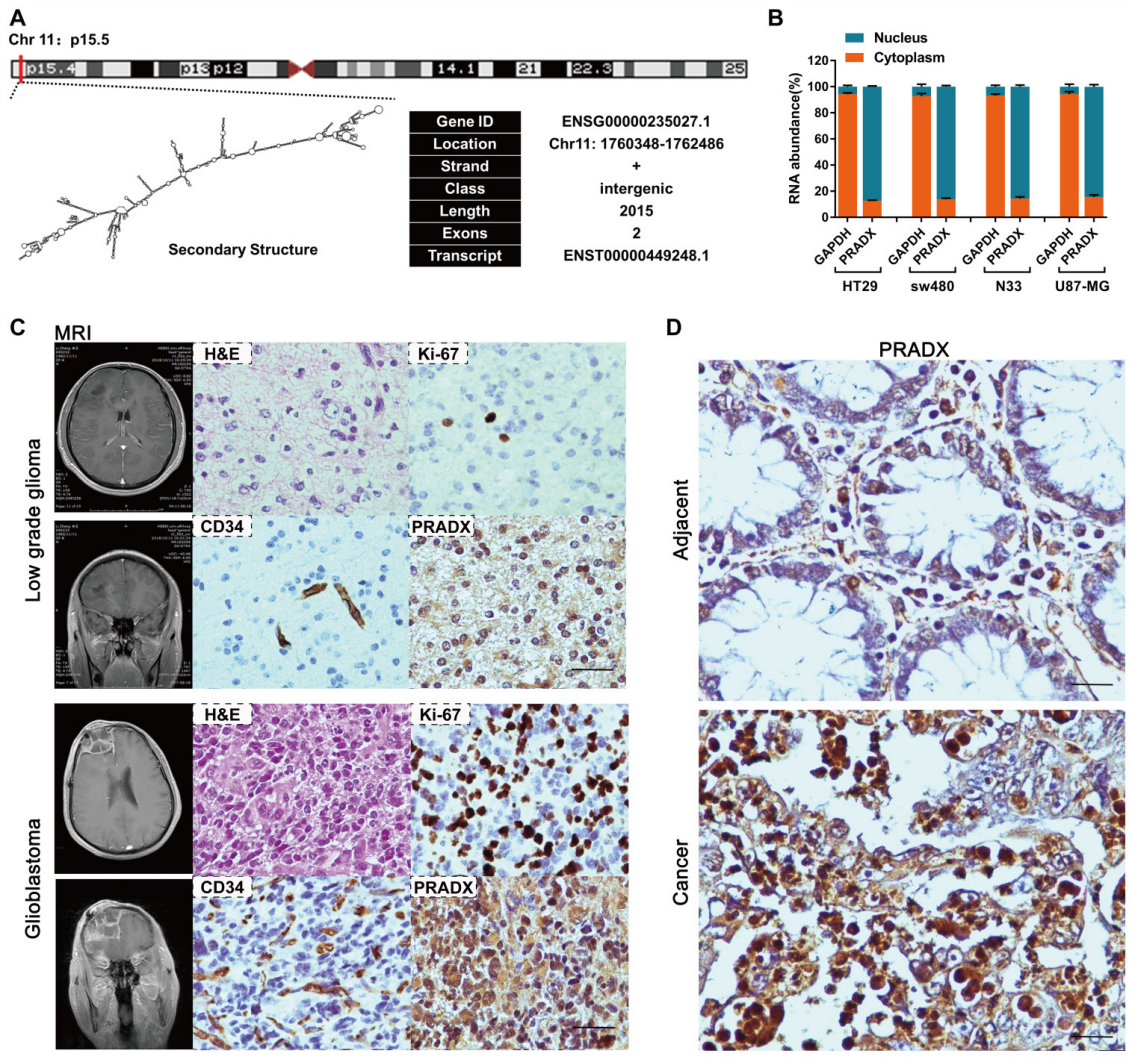
Identification of PRADX and its cellular localization in cancer tissues. **A** Genomic location, basic information and secondary structure (obtained from Lnc2Catlas database) of PRADX (ENST00000449248.1). **B** qRT-PCR analysis indicating the cytoplasmic/nuclear ratio of PRADX and GAPDH. **C** Representative MRI images of the low grade glioma and glioblastoma patients, and H&E staining, ICH and ISH images of the corresponding tissues. Scale bar, 40 μm. **D** Representative ISH images indicating PRADX expression in colon adenocarcinoma and adjacent normal tissues. Scale bar, 20 μm.

Lnc2Catlas database suggested that PRADX is a novel transcript located in the forward strand of chromosome 11:1,760,348-1,762,486 (Figure 3A). To determine if the novel PRADX transcript is a lncRNA, we first identified its 5′ and 3′ ends as well as the full-length sequence by using 5′ and 3′ RACE PCR (Figure S1A-B). Then we used PRIDE database 29, Lee translation initiation sites 30, PhyloCSF 31, Bazzini small ORFs 32, and Coding Potential Calculator 2 33, and showed that PRADX is a lncRNA rather than a protein-coding transcript (Figure S1C). Furthermore, real-time RT-PCR analysis of the nuclear and cytoplasmic fractions of GBM and COAD cells showed that PRADX is mainly located in the nucleus (Figure 3B). Additionally, ISH assays using 20 low grade glioma tissues, 22 GBM tissues, and 30 COAD and adjacent normal tissues further confirmed the predominant nuclear distribution of PRADX in tumor cells, and indicated its high expression in GBM and COAD (Figure 3C-D, Figure S1D).

### PRADX directly interacts with EZH2 protein via 1-500 bp 5' terminal sequence

**Figure 4:**
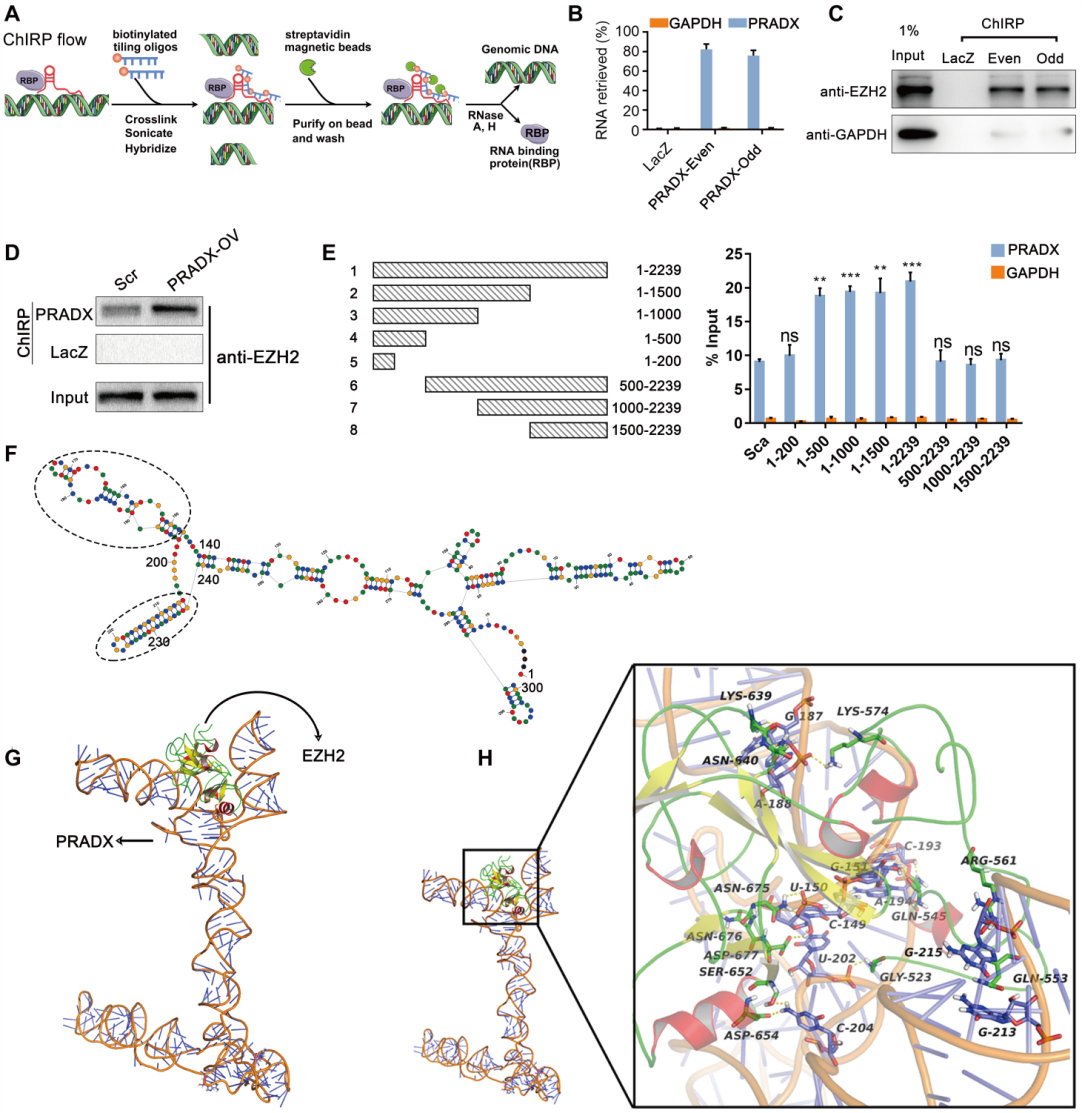
PRADX binds to EZH2 protein via the 5' terminal sequence in cancer cells. **A** ChIRP assay workflow for detecting the interaction between PRADX and EZH2. **B** Enrichment of PRADX in the pull-downs of both even and odd probe sets targeting PRADX relative to control LacZ probes in U87-MG cells, as detected by ChIRP assay. GAPDH was used as a negative control. **C** Enrichment of EZH2 protein in the pull-downs of both even and odd probe sets targeting PRADX relative to the control LacZ probes in U87-MG cells, as detected by ChIRP assay. **D** Enrichment of EZH2 protein in the pull-downs of PRADX probes relative to LacZ probes in U87-MG cells overexpressing PRADX (PRADX-OV) or control (Scr). **E** Mapping of the PRADX regions required for interaction with EZH2 (left). RIP-qPCR assays were performed using anti-EZH2 antibody in U87-MG cells overexpressing different PRADX segment constructs (right). GAPDH was used as a negative control. The values are represented as mean ± SD (n = 3). **P < 0.01, ***P < 0.001, ns, not statistically significant. **F** The secondary structure of PRADX (200-500 nt); oval dotted line indicated the Hairpin loop structure. **G** The tertiary structure of PRADX was docked with the EZH2 protein. **H** The intermolecular interaction details.

To investigate the role of PRADX in tumorigenesis, gene ontology analysis of PRADX co-expression cluster (obtained from Lnc2Catlas) was performed. Our results suggested that the expression of PRADX is associated with multiple biological processes, including cell cycle, DNA repair, and immune response (Table S5). Additionally, the ontology analysis revealed the PRADX co-expressed genes to be associated with histone modification, suggesting that the lncRNA might be implicated in tumorigenesis through epigenetic mechanisms (Figure S2A). Furthermore, as PRADX is mainly localized in the nucleus, we tried to identify if it interacts with EZH2, a validated critical RNA-binding protein 34, using ChIRP assays (Figure 4A). Twelve oligonucleotide probes targeting PRADX were divided into an even and odd set to create two independent groups of probe sets. Compared to LacZ probes (non-targeting control), both even and odd probe sets pulled down most of the PRADX from U87-MG human glioblastoma cells (Figure 4B). In addition, the analysis of retrieved protein validated the significant enrichment of EZH2 in the pull-downs of both even and odd probe sets targeting PRADX relative to control LacZ probes in U87-MG cells (Figure 4C). Furthermore, U87-MG cells overexpressing PRADX retrieved higher amount of EZH2 protein during the pull-down than those transfected with scramble (Figure 4D). These results suggest that PRADX directly interacts with EZH2. To characterize the exact binding site of EZH2 on PRADX, we generated a series of PRADX constructs with different deletion mutants and transfected these into U87-MG cells (Figure S3A), followed by RIP assays. The results showed that anti-EZH2 antibody retrieved significantly higher amounts of PRADX from cells transfected with 1-500, 1-1000, 1-1500 and 1-2239 bp constructs than those transfected with scramble (Figure 4E). In addition, 1-500 bp region of PRADX was found to be necessary and sufficient for EZH2 binding.

To understand how PRADX interacts with EZH2 at molecular level, we analyzed the structural data related to PRADX-EZH2 interaction. Based on the results of RIP assays, the potential EZH2-interaction sequence of PRADX (200-500 nt) was used for predicting the secondary structure using RNA Structure online tool (https://rna.urmc.rochester.edu/RNAstructureWeb/). The tool predicted two hairpin loop structures between 340-440 nt, which may correspond to the protein binding domain (Figure 4F). Furthermore, we predicted the corresponding three-dimensional RNA structure with 3dRNA v2.0 35 and optimized it in aqueous solution using Gromacs5.0 kinetics software (Figure 4G). Then, the tertiary structure of PRADX was docked with the EZH2 protein, thereby constructing the EZH2-PRADX protein nucleic acid complex (Figure 4G). The docking results revealed 5 potential complexes with high binding affinity, and the optimal docking score was -307.02 Kj/mol (Figure S4A-B). Notably, in all the 5 binding conformations, EZH2 binding was mostly observed between the two hairpin structures (340~440 nt) of PRADX, forming a "U" type chimeric structure. Next, we analyzed the intermolecular interactions of the optimal binding conformations. We observed the formation of strong hydrogen bonds between the key residues of EZH2, such as GLY523, GLN545, GLN553 and the bases in the 340-440 nucleotide region of PRADX, and formation of multi-hydrogen bonds particularly between GLN545, ASN640, and ASN675 residues and G151, C193, G187, and U150 bases (Figure 4H, Figure S4C). It is known that all RNA bases, the 2' OH, and the phosphodiester backbone can form hydrogen bonds and electrostatic interactions with proteins 36. By analyzing the electrostatic surface of EZH2 protein, we found that the binding surface of EZH2 was mostly a positively charged region, which interacted strongly with the negatively charged region of the outer oxygen atoms of PRADX spiral structure, and led to a stable binding between EZH2 and PRADX (Figure S4D). These data suggest that good spatial matching, hydrogen bonds and electrostatic interactions are key to PRADX-EZH2 interaction.

### PRADX recruits the PRC2/DDX5 complex

**Figure 5:**
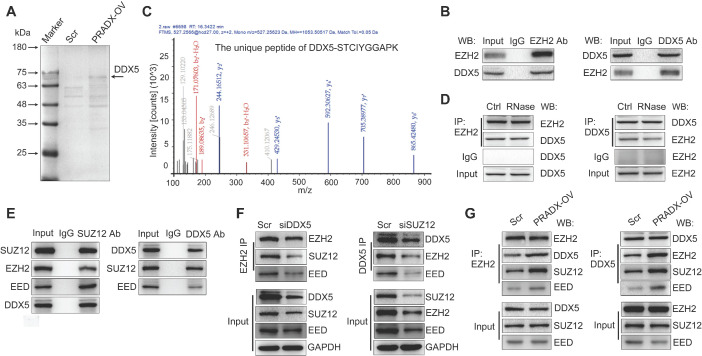
PRADX recruits PRC2/DDX5 complex. **A** Detection of EZH2-associated candidate proteins by Co-IP assays in PRADX or scramble overexpressing U87-MG cells. **B** Co-IP assays were performed with anti-EZH2 or anti-DDX5 antibody, followed by immunoblotting with EZH2 or DDX5 antibodies. **C** Characterization of the EZH2 immunoprecipitated band by mass spectrometry. **D** Co-IP assays were performed with anti-EZH2 or anti-DDX5 antibody, followed by immunoblotting with EZH2 or DDX5 antibodies after RNase treatment. **E** Co-IP assays were performed with anti-SUZ12 or anti-DDX5 antibody, followed by immunoblotting with SUZ12, EZH2, EED or DDX5 antibodies. **F** Co-IP assays were performed with anti-EZH2 or anti-DDX5 antibody, followed by immunoblotting with SUZ12, EZH2, EED and DDX5 antibodies after DDX5 or SUZ12 knockdown. **G** Co-IP assays were performed with anti-EZH2 or anti-DDX5 antibody, followed by immunoblotting with SUZ12, EZH2 EED, and DDX5 antibodies after PRADX overexpression. Scr: Scramble; PRADX-OV: PRADX overexpression.

To gain deeper insights into the mechanisms of PRADX-EZH2 interaction in tumorigenesis, we performed EZH2-Co-IP assay using U87-MG cells overexpressing PRADX or transfected with scramble. Analysis of the immunoprecipitated proteins by sodium dodecyl sulfate-polyacrylamide gel electrophoresis and Coomassie staining detected a distinct brighter band in the PRADX overexpression group compared to the control group (Figure 5A). Furthermore, MS analysis of the band identified 83 potential EZH2 binding proteins (Table S6); of these, DDX5, structure specific recognition protein 1 (SSRP1), protein phosphatase 2 scaffold subunit alpha (PPP2R1A), and heterogeneous nuclear ribonucleoprotein M (HNRNPM) proteins are known to be located in the nucleus and may be involved in histone modification or transcription 20, 37-39. Thus, we selected these 4 proteins for further validation, and Co-IP assays confirmed that only DDX5 bound specifically to EZH2 protein (Figure 5B, Figure S3C). MS analysis identified 3 unique peptides of DDX5 that were validated by peptide identification spectra (Figure 5C, Figure S3B). By using RIP assays, PRADX was not enriched by DDX5 (Figure S3G). Furthermore, RNase treatment before Co-IP did not affect the interaction between EZH2 and DDX5 (Figure 5D) As we know that protein can switch the spatial conformation once interaction with RNA 40, we hypothesized that EZH2 could change the spatial conformation after interacted with PRADX, increasing the binding affinity to other subunits of PRC2 complex and DDX5. To investigate whether DDX5 could interact with other core subunits of PRC2 complex, Co-IP assays were employed. The results showed that DDX5 can be Co-IP with SUZ12 and EED (Figure 5E). A previous study reported that DDX5 is involved in stabilizing the PRC2-mediated gene silencing 20. Thus, we performed Co-IP experiments using U87-MG cells following knocking down of DDX5 or SUZ12 genes (Figure S3D). Knockdown of DDX5 significantly reduced Co-IP of endogenous EZH2 with other core PRC2 subunits, whereas, knockdown of SUZ12 significantly reduced Co-IP of endogenous DDX5 with other core PRC2 subunits (Figure 5F). Furthermore, PRADX overexpression significantly increased the Co-IP of endogenous EZH2 or DDX5 with other core PRC2 subunits (Figure 5G). Collectively, PRADX recruits PRC2/DDX5 complex by interacting with EZH2, suggesting that it may be involved in histone modification.

### PRADX suppresses UBXN1 expression via PRC2/DDX5 complex and promotes the nuclear transport of NF-κB

**Figure 6:**
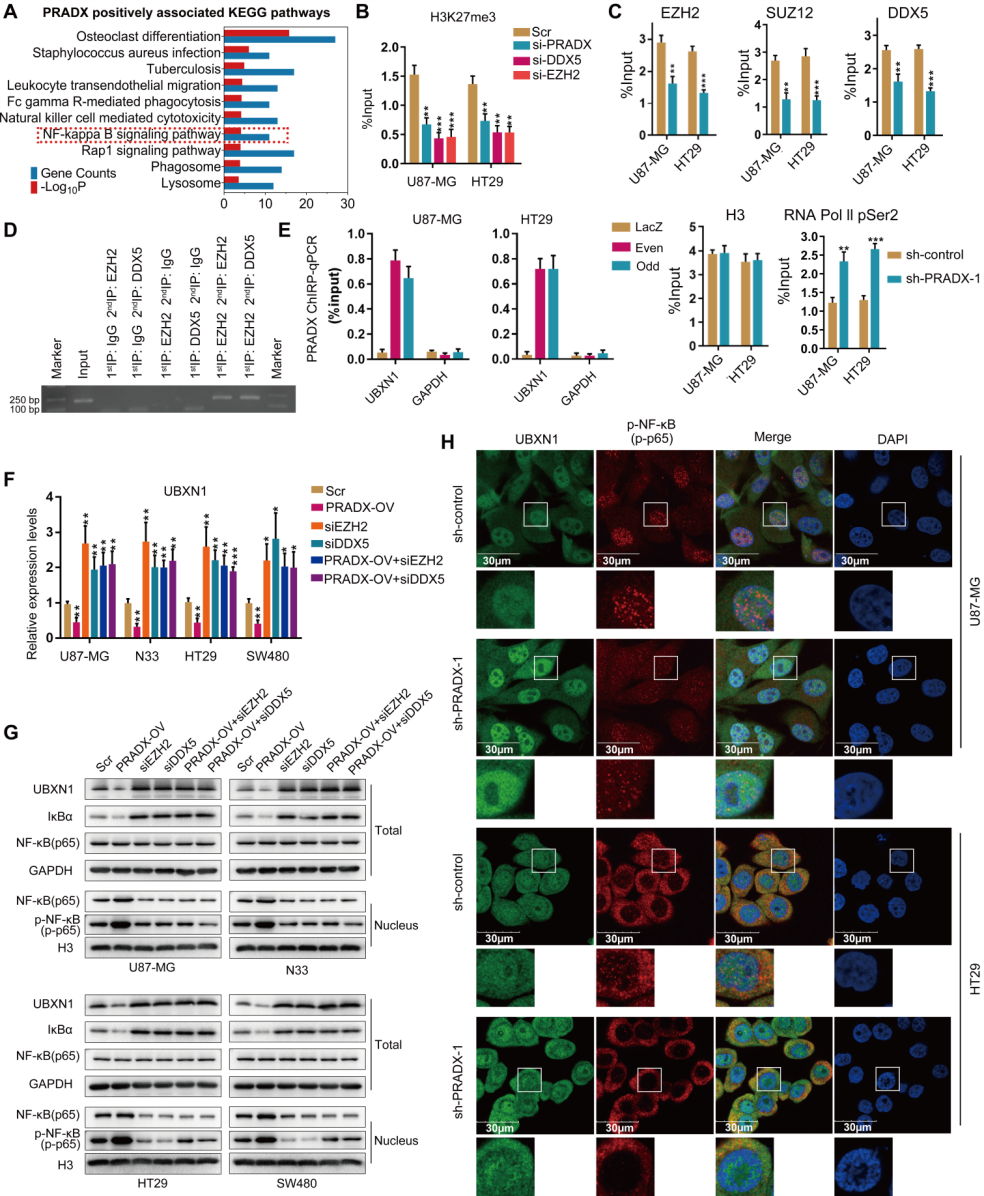
PRADX regulates NF-κB activity by recruiting PRC2/DDX5 in cancer cells. **A** Top 500 overlapping genes associated with PRADX expression in GBM and COAD cohorts were screened and subjected to KEGG pathway analysis. **B** ChIP-qPCR results showing H3K27Me3 occupancy levels in the UBXN1 promoter region in PRADX, DDX5 or EZH2 knockdown and scramble groups. **C** ChIP-qPCR showing occupancy levels of EZH2, DDX5, SUZ12, RNA Pol II pSer2 and H3 at UBXN1 promoter region in PRADX knockdown and control groups. **D** ChIP-reChIP assays showing co-occupancy of DDX5 and EZH2 at UBXN1 promoter region. **E** ChIRP assays showing enrichment of UBXN1 promoter fragment pulled-down by even and odd probe set groups targeting PRADX compared to control LacZ probes in U87-MG and HT29 cells. **F** The mRNA levels of UBXN1 were detected in PRADX overexpression, EZH2 knockdown, DDX5 knockdown, PRADX overexpression plus EZH2 knockdown, PRADX overexpression plus DDX5 knockdown or scramble groups by RT-qPCR. **G** Western blotting results showing the total protein levels of UBXN1, Iκα and NF-κB and the nuclear protein levels of NF-κB and p-NF-κB upon PRADX overexpression and/or EZH2 or DDX5 knockdown or scramble groups. **H** Immunofluorescence staining showing UBXN1 and p-NF-κB expression in PRADX knockdown or scramble groups. DAPI was used to stain the nuclei. Scale bar, 30 μm. The values in **C, D, E** and** F** are represented as mean ± SD (n = 3). *P < 0.05, **P < 0.01, ***P < 0.001.

To explore the biological functions of PRADX, genes positively associated with PRADX expression in GBM and COAD cohorts were screened. Top 500 overlapping genes were subjected to GO and KEGG pathway analysis. Both the analysis revealed that PRADX expression significantly correlated with inflammatory and immune responses, as well as NF-κB related annotations and signaling pathway (Figure 6A, Table S7). Similarly, GSEA indicated that patients with high PRADX expression were enriched for NF-κB related gene sets, including positive regulation of NF-κB transcription factor activity (Figure S5, Table S8). The gene set variation analyses further confirmed the positive relationship between PRADX expression and NF-κB related gene sets (Figure S2B). Because UBXN1 negatively regulates the NF-κB activity by inhibiting IκBα degradation 41, and is a target for EZH2 mediated H3K27me3 according to our previous study 25, we evaluated if the chromatin state of the UBXN1 promoter region is regulated by PRADX and PRC2/DDX5 complex. ChIP-qPCR analysis showed that knockdown of PRADX, EZH2 or DDX5 reduced H3K27me3 enrichment at UBXN1 promoter region (Figure 6B, Figure S3D-E). Moreover, PRADX knockdown reduced EZH2, DDX5, or SUZ12 occupancy at UBXN1 promoter, whereas, increased the occupancy of RNA Pol II pSer2, indicating transcription extension (Figure 6C, Figure S3F). ChIP-reChIP assays were furtherly employed to clarify the occupancy of DDX5 and PRC2 complex in the promoter of UBXN1 (Figure 6D). To validate whether PRADX directly interacts with UBXN1 promoter, ChIRP assays were performed using the oligonucleotide probes targeting PRADX in even and odd sets followed by amplification of UBXN1 promoter sequences. Compared to the LacZ groups, both the probe set groups showed substantial amplification of UBXN1 promoter region, however no amplification was observed for GAPDH (Figure 6E). Furthermore, investigation of UBXN1 mRNA levels in GBM and COAD cell lines demonstrated that PRADX overexpression mediated decrease of UBXN1 expression was abrogated by EZH2 or DDX5 knockdown (Figure 6F). Consistently, the requirement of PRC2/DDX5 in PRADX-mediated target gene repression was further supported by reversal of PRADX-mediated decrease of UBXN1 protein levels in EZH2- or DDX5-depleted cells (Figure 6G). Collectively, PRADX promotes H3K27me3 of the UBXN1 promoter region and decreases its expression via recruiting the PRC2/DDX5 complex. Besides above, we also detected some target genes of PRC2 complex and identified the general regulatory mechanism mediated by PRADX and DDX5 (data not shown).

UBXN1, a family member of UBX domain containing proteins, colocalizes with ubiquitin and blocks the canonical NF-kB pathway by inhibiting IκBα degradation 41. Western blot analysis showed that tumor cells overexpressing PRADX had reduced levels of UBXN1, which in turn decreased IκBα levels and increased the nuclear levels of NF-κB and p-NF-κB. However, knocking down EZH2 or DDX5 in tumor cells increased IκBα levels and decreased the nuclear levels of NF-κB and p-NF-κB. Furthermore, PRADX overexpression mediated decrease in IκBα and increase in nuclear NF-κB and p-NF-κB levels was abrogated by EZH2 or DDX5 knockdown (Figure 6G). Knockdown of PRADX had the opposite effects (Figure S6A-B). In addition, immunofluorescence staining revealed that PRADX knockdown upregulated UBXN1 expression and decreased nuclear p-NF-κB levels (Figure 6H). The expression level of UBXN1 via IHC assays was interrogated in GBM and COAD tissues. As shown in Figure S7A-D, GBM and COAD tumor tissues presented the low expression of UBXN1, compared with LGG and adjacent tissues of COAD. Furthermore, the quantitative data of PRADX and UBXN1 IHC staining in same tissues were used to measure the correlation. As shown in Figure S7E-F, the Pearson correlation coefficients were statistically significant, indicating that the expression levels of PRADX were negatively correlated with UBXN1 in GBM and COAD tissues. These results suggest that PRADX promotes the nuclear transport of NF-κB by suppressing UBXN1 expression.

### PRADX knockdown inhibits tumor growth and tumorigenesis, and prolongs the survival of tumor bearing mice

**Figure 7:**
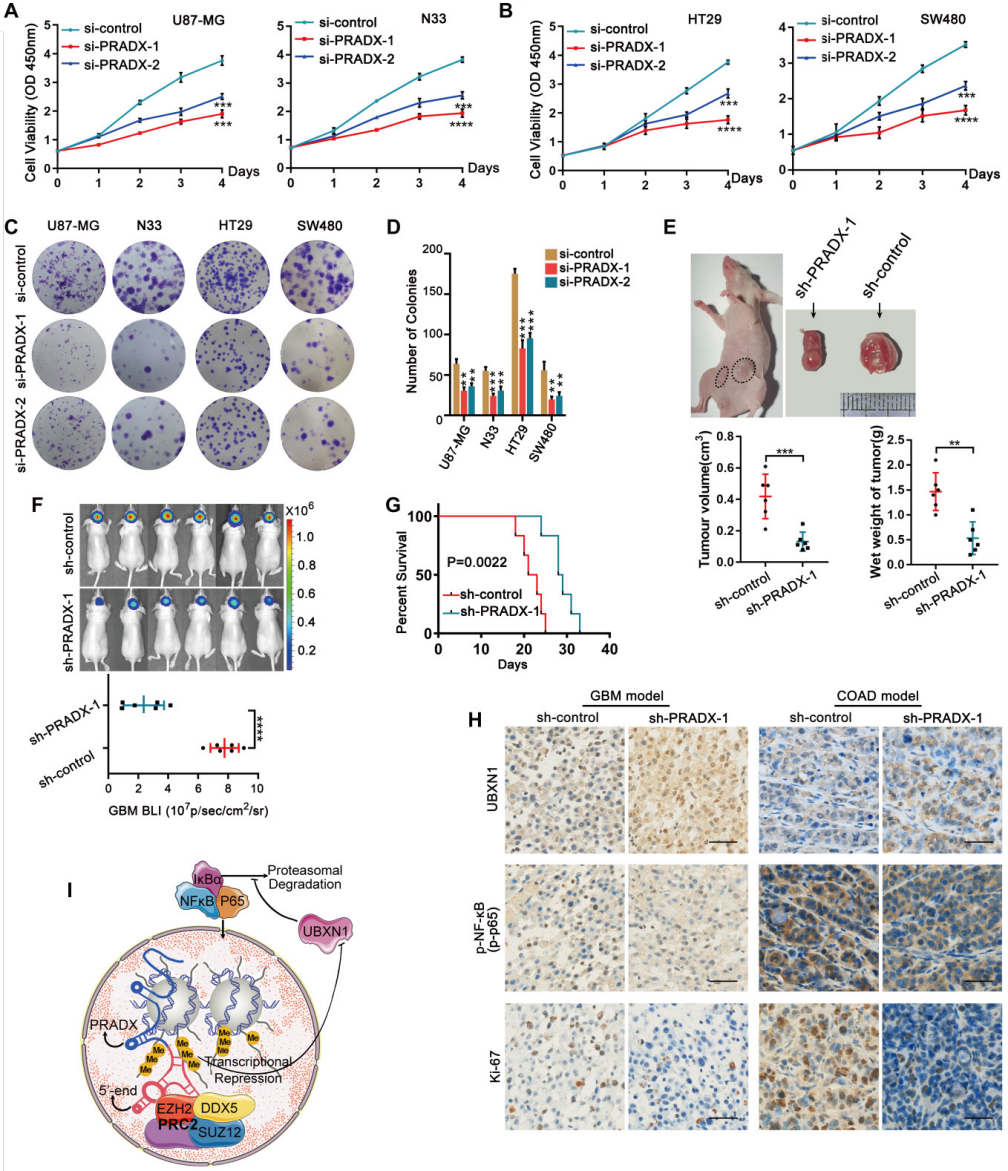
PRADX promotes tumor cell growth and tumorigenesis. **A**-**B** CCK-8 assays showing the effect of PRADX knockdown on the viability of U87-MG, HT-29, N33 and SW480 cells. **C**-**D** Clonogenic assays showing the effect of PRADX knockdown on U87-MG, HT-29, N33, and SW480 cells. Representative images and the average number of colonies are shown. **E** Nude mice were subcutaneously injected with PRADX knockdown or control HT29 cells. Quantification of tumor volume and wet weight is shown. **F**-**G** Nude mice were orthotopically injected with PRADX knockdown or control U87-MG cells. Quantification of bioluminescent imaging signal intensities and Kaplan-Meier survival curve of nude mice are shown. **H** Immunohistochemistry of tumor tissues from xenograft models showing UBXN1, p-NF-κB and Ki-67 expression in PRADX knockdown and scramble groups. Scale bar, 40 μm. **I** Scheme showing PRADX-mediated recruitment of PRC2/DDX5 complex and regulation of NF-κB activity. The values in **A, B, D, E** and** F** are represented as mean ± SD (n = 3). *P < 0.05, **P < 0.01, ***P < 0.001, ****P < 0.0001.

Knockdown of PRADX significantly inhibited the viability of GBM and COAD cells (Figure 7A-B). Furthermore, clonogenic assays demonstrated that PRADX knockdown significantly inhibited the growth of cancer cells compared to siRNA control (Figure 7C-D). To investigate the effect of PRADX knockdown *in vivo*, we developed mouse subcutaneous COAD models using PRADX-knockdown or control HT29 cells. Three weeks after implantation, the tumors were removed and analyzed. We found that the volume and wet weight of the tumors were significantly lower in the PRADX-knockdown group than in the control group (Figure 7E). Additionally, we developed orthotopic GBM models using PRADX-knockdown or control U87-MG cells. Bioluminescence images, captured after 2 weeks of implantation revealed significantly smaller tumors in the PRADX-knockdown group than in the control group (Figure 7F). Moreover, the mice in the PRADX-knockdown group had prolonged survival than those in the control group (Figure 7G). Furthermore, the tumors from the PRADX-knockdown group of COAD and GBM models had higher levels of UBXN1 and lower levels of p-NF-κB and Ki67 than that of the control groups (Figure 7H). These data suggest that PRADX is an oncogenic lncRNA and a potential therapeutic target in glioblastoma and colon adenocarcinoma. A schematic diagram showing PRADX-mediated recruitment of PRC2/DDX5 complex and regulation of NF-κB activity is shown in Figure 7I.

## Discussion

The unceasing advances in functional genomics have led to the discovery of thousands of lncRNAs. Their biological roles have been established as *cis* or *trans* regulators of transcription, modulators of mRNA processing, in transcriptional regulation and protein activity, and organization of nuclear domains 42, 43. However, the exact mechanisms of actions of only a few lncRNAs have been characterized and established 44, 45. Recently, research on lncRNA has attracted the attention of various cancer researchers. To investigate the aberrant expression pattern of lncRNAs in tumors, we analyzed whole transcriptome of TCGA pan-cancer datasets and revealed the differential expression profiles of lncRNAs in 12 tumors and paired normal tissues. Further, we identified a differentially expressed transcript, ENST00000449248.1, also termed as PRADX, which may serve as a potential prognostic indicator in GBM and COAD. PRADX was demonstrated to be a novel lncRNA with predominant nuclear distribution and high expression in GBM and COAD.

The majority of the lncRNAs are predominantly located in the nucleus, which indicates their possible regulatory role in transcription. In addition, a few lncRNAs are present in both cytoplasm and nucleus 46, 47. Thus, most of the lncRNAs may have protein-binding potential. EZH2 was the first protein to be used as an RNA binding protein in RIP-seq 48. A recent study demonstrated that the PRC2 subunit, EZH2 has the highest binding affinity towards RNA molecule and is somewhat promiscuous 49. However, this does not exclude the possibility of lncRNAs specifically interacting with EZH2. In this study, we not only provide experimental evidence for direct and specific interactions of PRADX and EZH2, but also show that the binding mechanism is based on three distinct aspects, i.e., good spatial matching, hydrogen bonds and electrostatic interaction. Additionally, multiple studies have shown that the higher-order structure of RNA and its interactions have multiple functions 50, 51. Hence, identifying RNA structures that are involved in gene regulation and function is of utmost importance for understanding the underlying biological mechanisms.

Proteins function mainly by interacting with other proteins. Based on Co-IP and MS analysis, we found that DDX5 interacts with EZH2 and thus is recruited by PRADX together with other PRC2 core components. Moreover, DDX5 was shown to be dependent on PRADX-mediated UBXN1 suppression. Hence, DDX5 knockdown resulted in PRC2 degradation. In support of our observation, the helicase activity of DDX5 has been shown to stabilize PRC2-mediated gene silencing, by displacing the RNA-binding E3 ligase from HOTAIR 20. Thus, PRADX can result in gene silencing by recruiting PRC2/DDX5 complex; however, the role of DDX5 in the complex still needs further exploration. In the current study, PRADX suppressed UBXN1 transcription via H3K27me3 of the promoter and promotes the nuclear translocation NF-κB (Figure 7I). Activated NF-κB pathway, which is well-known as a dominant character of the inflammation and tumorigenesis of GBM and COAD 52-54, contributes to PRADX mediated tumor progression.

Although many lncRNAs are dysregulated in a tumor-specific manner, a few, including OIP5-AS1, TUG1, NEAT1, MEG3, and TSIX, synergistically dysregulate the cancer associated pathways in multiple tumor contexts 55. Our results indicated that PRADX activates NF-κB pathway by suppressing UBXN1 expression in both GBM and COAD cells, indicating that a single lncRNA may be used as a marker or therapeutic target against multiple tumors.

## Conclusions

We identified a novel cancer driver lncRNA, PRADX that recruits PRC2/DDX5 complex by interacting with EZH2 and promotes NF-κB activity via UBXN1 suppression, which in turn contributes to GBM and COAD tumorigenesis.

## Supplementary Material

Supplementary figures and tables.

## Abbreviations

lncRNA: Long noncoding RNA
PRC2: Polycomb repressive complex 2
TCGA: The Cancer Genome Atlas
GBM: Glioblastoma
COAD: Colon adenocarcinoma
SUZ12: Suppressor of zeste 12 homolog
EZH2: Enhancer of zeste homologue 2
EED: Embryonic ectoderm development protein
H3K27me3: Trimethylation of histone H3 at lysine 27
DDX5: DEAD box protein 5
CCK-8: Cell Counting Kit 8
Co-IP: Co-Immunoprecipitation
MS: Mass spectrometry
ChIP: Chromatin Immunoprecipitation
RT-qPCR: Real-time quantitative reverse-transcription PCR
RIP: RNA Immunoprecipitation
ISH: RNA *in situ* hybridization
ChIRP: Chromatin isolation by RNA purification
IHC: Immunohistochemistry
H&E: Hematoxylin and eosin
ANOVA: Analysis of variance
GO: Gene Ontology
GSEA: Gene set enrichment analysis
SSRP1: Structure specific recognition protein 1
PPP2R1A: Protein phosphatase 2 scaffold subunit alpha
HNRNPM: Heterogeneous nuclear ribonucleoprotein M
