# Supplementary material for: LncRNA PRADX-mediated recruitment of PRC2/DDX5 complex suppresses UBXN1 expression and activates NF-κB activity, promoting tumorigenesis
Source: Theranostics. 2021 Mar 4;11(9):4516–30. doi: 10.7150/thno.54549 (PMC7977445; doi:10.7150/thno.54549)
Supplement: Supplementary file 1 — Supplementary figures and tables. [file thnov11p4516s1.pdf]

Supplementary materials

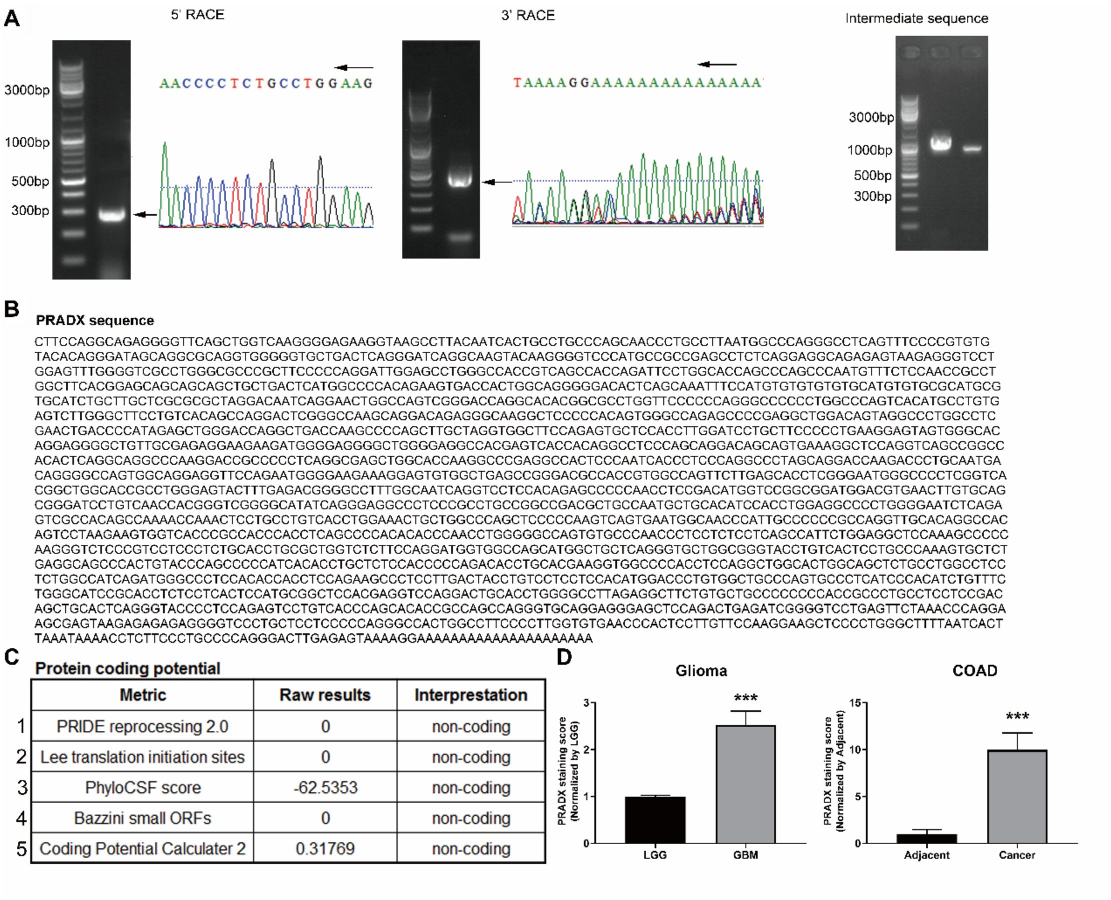

Figure S1 PRADX is a novel lncRNA in cancer cells. **A** Representative images of PCR products from the 5'RACE (left panel), 3'RACE (middle panel), or intermediate sequence (right panel). The major PCR product is marked by an arrow, and the sequence of the PCR product is shown beside it with an arrow indicating the boundary of PPADX sequence. **B** The full-length sequence of PRADX. **C** The coding potential was analyzed for the PRADX sequence by 5 different means. **D** Quantification of PRADX expression was measured by ImageJ software. ***$P < 0.001$.

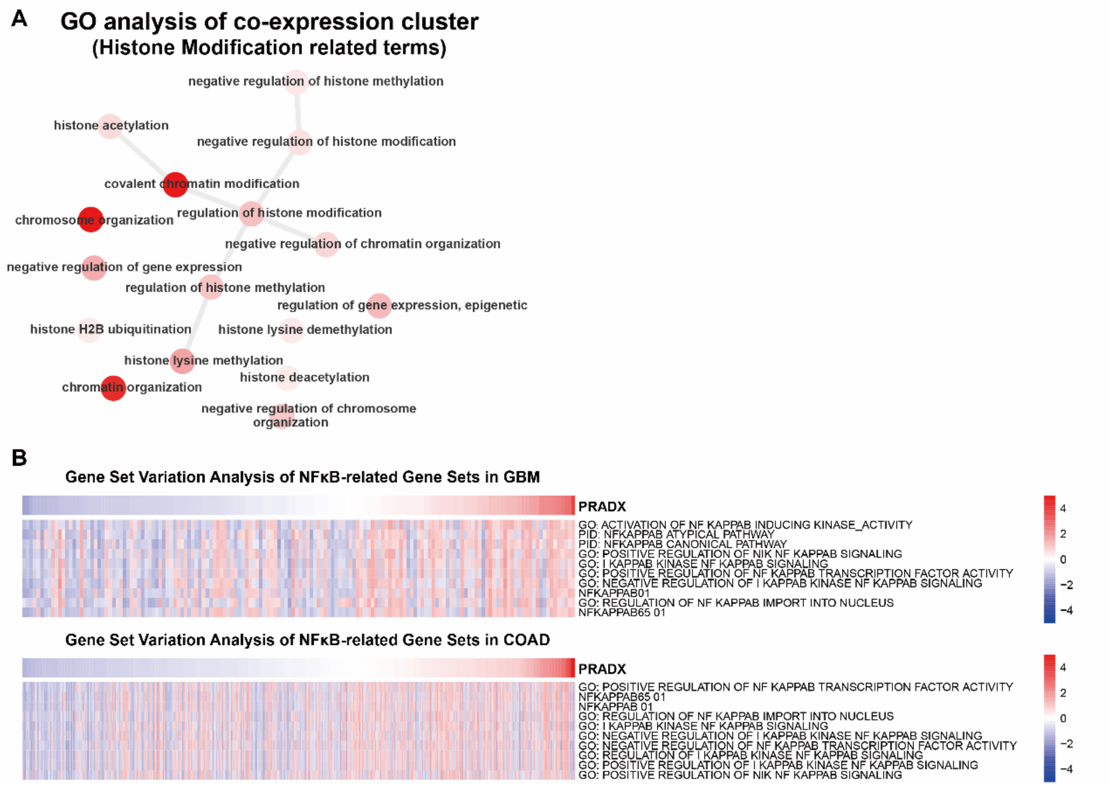

Figure S2 The gene ontology (GO) and gene set variation analyses of PRADX co-expression cluster. **A** GO analysis of Histon Modification related terms. **B** Gene set variation analyses showed that NF-κB related genes highly expressed in GBM or COAD smples with high PRADX expression.

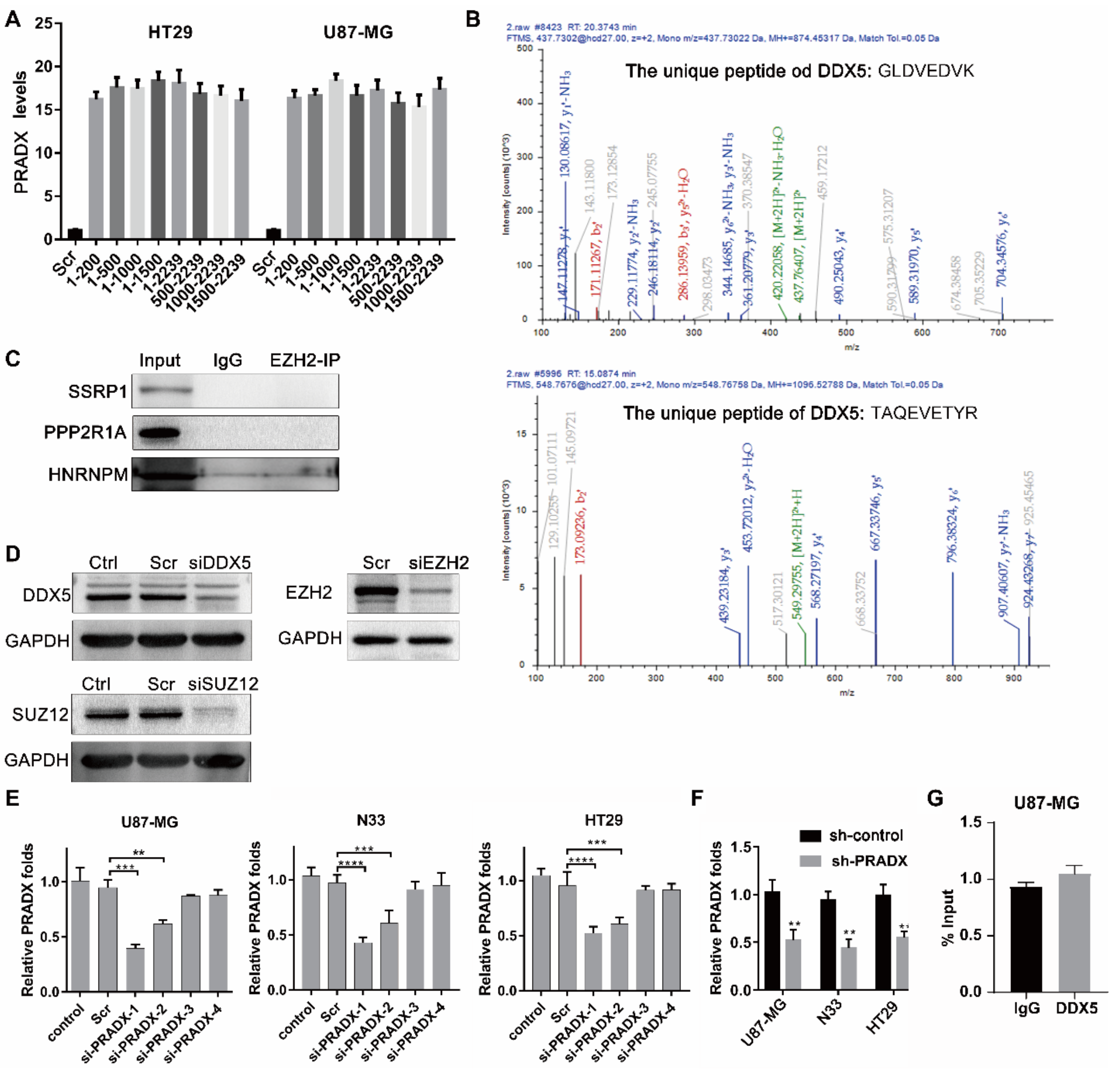

Figure S3 EZH2 interacts with DDX5. **A** qRT-PCR analysis showing the PRADX levels in HT29 and U87-MG cells transfected with indicating PPADX fragments. **B** Unique peptides of DDX5 were validated by peptide identification spectra. **C** Western blot analysis showed that SSRP1, PPP2R1A or HNRNPM do not bind specifically to EZH2. **D** Western blot analysis showed the efficiency of knocking down DDX5, SUZ12 and EZH2. qRT-PCR analysis showed the efficiency of knocking down PRADX with different siRNAs (**E**) or lenti-shRNA (**F**) in indicated cell lines. **G** RIP assays were utilized to verify that PRADX did not interact with DDX5.

A

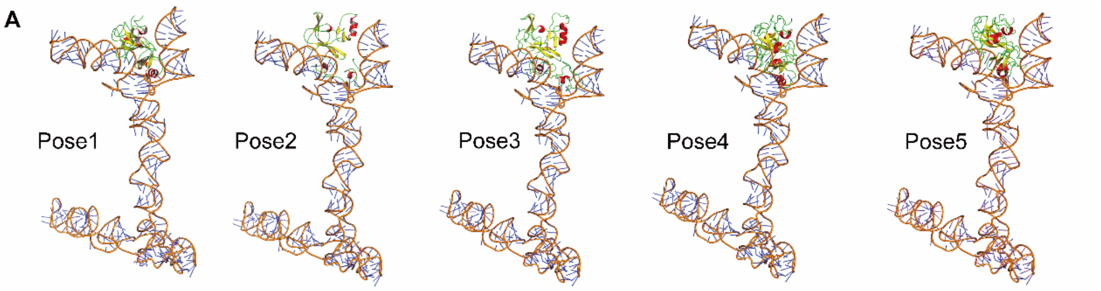

B

Complex	Docking Score / kJmol-1
Pose1	-307.02
Pose2	-273.62
Pose3	-266.21
Pose4	-264.05
Pose5	-262.03

C

RUN	Key Residue	Contacts	Distance	Angle DHA
1	GLY523	H04---OP2:U202	2.2	112.7
2	GLN545	O---H10:G151	2.6	127.9
	GLN545	OE1---H9:G151	2.4	118
	GLN545	H06---H10:C193	2.2	140.9
3	GLN553	H06---OE1:G213	1.9	99.8
4	ARG561	H01---O5:G215	2.7	108.9
5	LYS574	H01---OP2:A188	2.5	99.7
6	LYS639	H01---O4:G187	1.4	138.5
7	ASN640	H05---O2:G187	1.8	152.4
	ASN640	H05---O3:G187	2	108.8
8	SER652	OG---H08:C204	2.2	125.1
9	ASP654	OD2---H10:C204	2.2	174.5
10	ASN675	H01---OP2:U150	2.5	107.4
	ASN675	H05---O3:U150	1.9	96
11	ASN676	H02---OP1:C149	2	144.3
12	ASP677	OD2---H06:C149	2.3	135.6

D

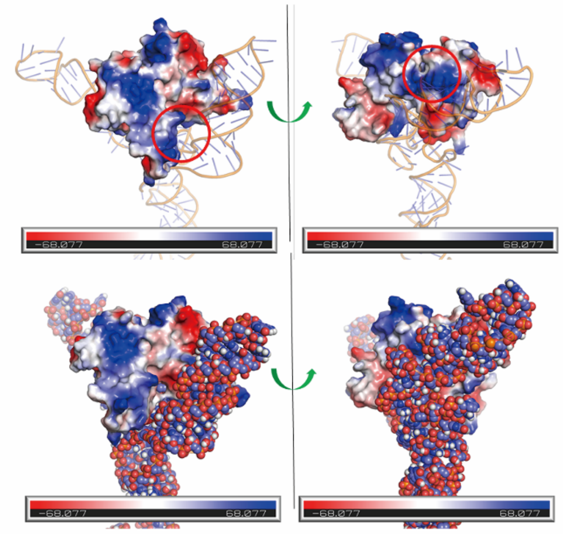

Figure S4 Molecular simulation study of PRADX and EZH2 (**A**) Top 5 complex conformations from molecular docking. (**B**) Docking Score of top 5 complex conformations. **C** Analysis of the hydrogen bond interaction between key residues in the binding site of EZH2 protein and PRADX. **D** Electrostatic surface (the up two graghs) and electrostatic matching (the down two graghs) of PRADX-EZH2.

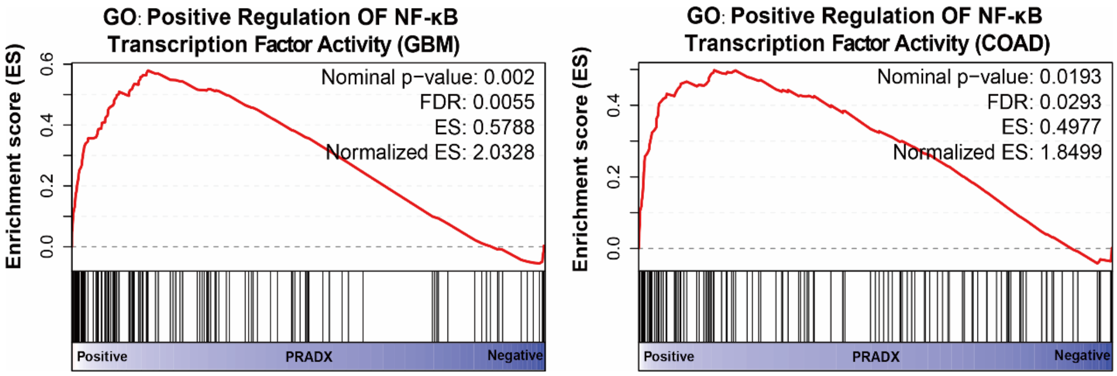

Figure S5 GSEA analysis showed that GBM and COADpatients with high PRADX expression were enriched for NF-κB related gene sets.

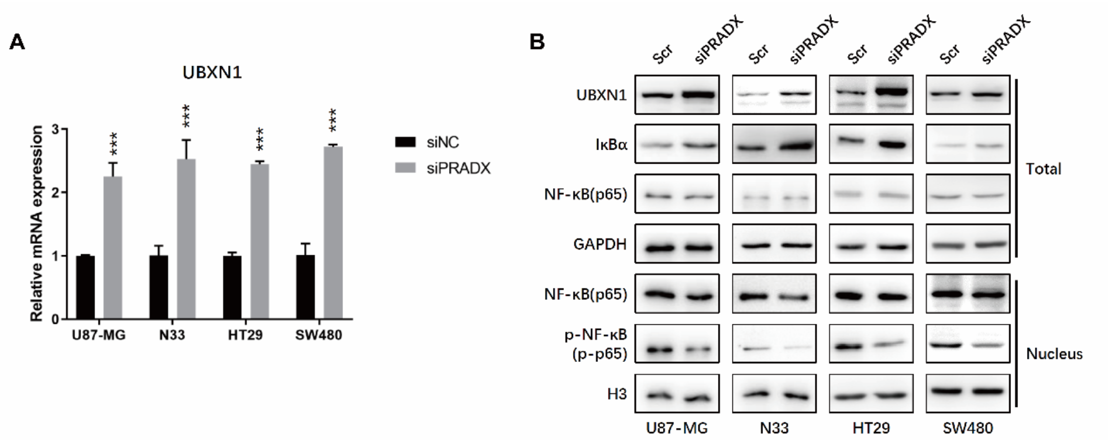

Figure S6 The expression of UBXN1 was regulated by PRADX. **A** The mRNA levels of UBXN1 were detected in PRADX knockdown or scramble groups by RT-qPCR. **B** Western blotting results showing the total protein levels of UBXN1, Iκα and NF-κB and the nuclear protein levels of NF-κB and p-NF-κB upon PRADX knockdown or scramble groups.

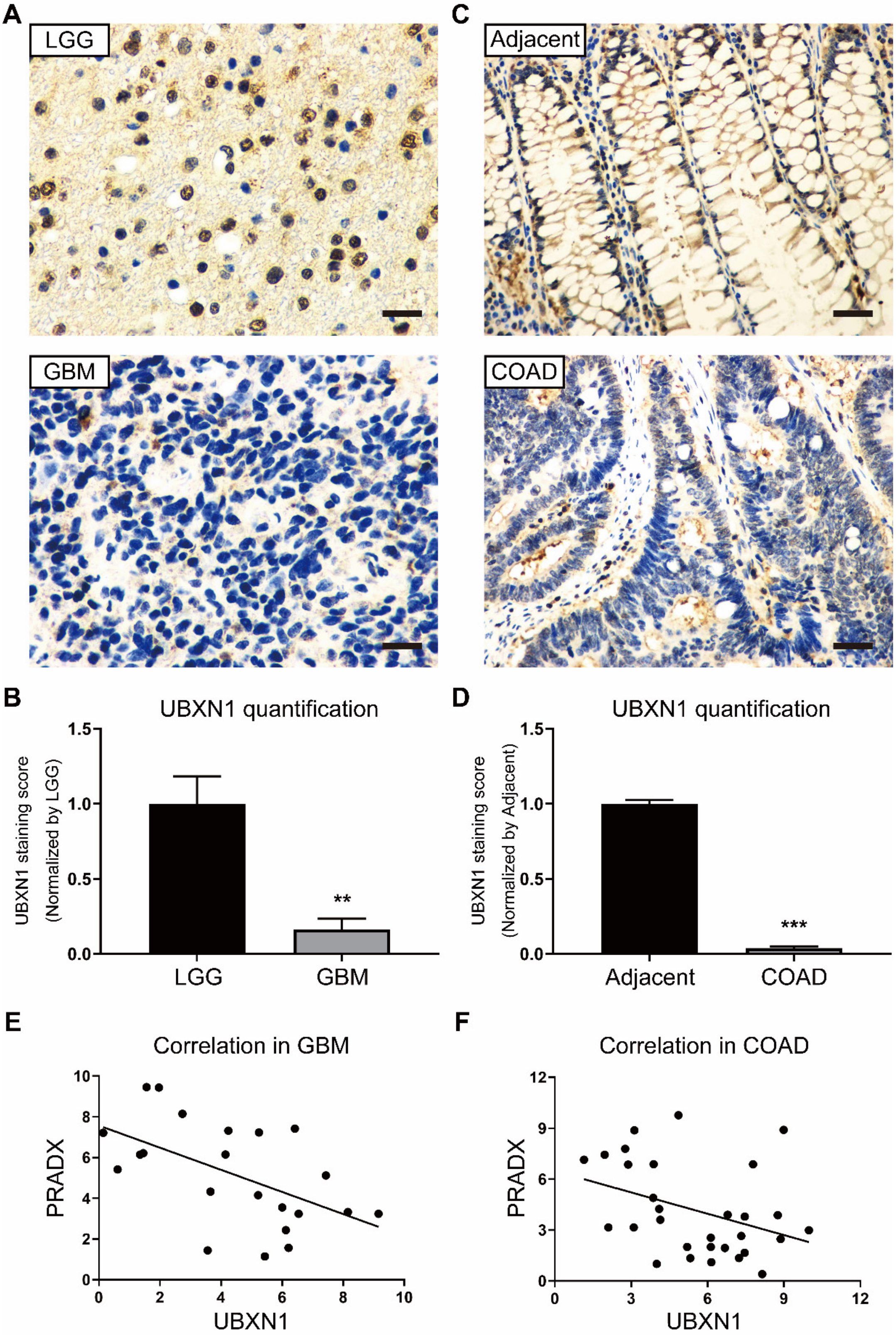

Figure S7 Identification of UBXN1 in glioma and COAD tissues. IHC assays were performed to analyze the expression of UBXN1 in LGG (**A**), GBM (**A**), adjacent (**C**),

and COAD cancer (**C**) tissues. Quantitative analysis was measured using ImageJ software (**B and D**). Scale bar, 20 μm. The quantitative data of PRADX and UBXN1 IHC staining in same tissues were used to verify the significant correlation (GBM: r = -0.55, *P* value = 0.008; COAD: r = -0.37, *P* value = 0.045) (**E-F**).

Table S1 Information of the RT-qPCR primers, ISH-probes, ChIRP-probs, antibodys and siRNA sequence.

Table S2 The differential expressed lncRNAs between tumor and paired adjacent tissues of 12 cancer types.

Table S3 The shared upregulated lncRNAs among 12 cancer types, compared to the matched normal tissues.

Table S4 The univariate COX regression analysis of top 12 lncRNAs in TCGA Pan-cancer Atlas.

Table S5 Gene Ontology analysis of co-expression cluster in TCGA Pan-cancer Atlas.

Table S6 Proteins in the distinct band identified by Mass Spectrometry.

Table S7 PRADX positively associated Gene Ontology and KEGG terms in both GBM and COAD.

Table S8 Gene Set Enrichment Analysis for PRADX high expression patients in GBM.
